# Treatment response to intravitreal bevacizumab in small pigmented choroidal lesions with subretinal fluid

**DOI:** 10.1186/s12886-019-1108-z

**Published:** 2019-05-03

**Authors:** Junwon Lee, Hee Jung Kwon, Min Kim, Christopher Seungkyu Lee, Sung Chul Lee

**Affiliations:** 1 Department of Ophthalmology, Eye and ENT Hospital, Severance Hospital, Institute of Vision Research, Yonsei University College of Medicine, 50-1 Yonsei-ro, Seodaemun-gu, Seoul, 03722 South Korea; 2 Department of Ophthalmology, CHA Bundang Medical Center, CHA University, 59 Yatap-ro, Bundang-gu, Seongnam-si, Gyeonggi-do 13496 South Korea; 3 Department of Ophthalmology, Institute of Human Barrier Research, Gangnam Severance Hospital, Yonsei University College of Medicine, 211 Eonju-ro, Gangnam-gu, Seoul, 06273 South Korea

**Keywords:** Bevacizumab, Subfoveal fluid, Small pigmented choroidal lesion, Transpupillary thermotherapy, Melanoma

## Abstract

**Background:**

To describe the effects of intravitreal bevacizumab injection (IVB) and/or transpupillary thermotherapy (TTT) in the treatment of small pigmented choroidal lesions with subfoveal fluid (SFF), and to investigate prognostic value of the therapeutic response in future tumor growth.

**Methods:**

Retrospective chart review of 19 patients, who were diagnosed with choroidal neovascularization (CNV)-free small pigmented choroidal lesions and treated with IVB and/or TTT, was performed.

**Results:**

Complete resolution of SFF was achieved in two eyes (2/14; 14.3%) after IVB, and in three eyes (3/4; 75%) after TTT. Best corrected visual acuity was improved in two eyes (2/9; 22%) after IVB, and in three eyes (3/4; 75%) after TTT. Among five patients who underwent TTT after IVB, four patients (4/5; 80%) demonstrated additional advantage. All IVBs could not reduce tumor sizes. Rather, tumor growth was detected in seven out of 14 eyes (7/14; 50%) that underwent IVB. None of the patients who underwent TTT showed tumor growth. The lack of treatment response to IVB was suggestive of malignancy, as most small pigmented lesions that had no response to IVB showed tumor growth (86%, *p* = 0.010).

**Conclusion:**

IVB was not effective in reducing tumor size and subfoveal fluid in small pigmented choroidal lesions. Therapeutic response to IVB can be used as an indicator between melanoma and nevus in small pigmented choroidal lesion.

## Background

Small pigmented choroidal lesions may be choroidal nevus or choroidal melanoma. It is very important to discriminate between benign choroidal nevus and small malignant choroidal melanoma; however, their differential diagnosis is not always easy. In addition, choroidal nevus is a subject of interest due to its possible malignant transformation into a melanoma [1], and thus, careful and continuous observation may be required.

Shields et al. analyzed 2514 consecutive choroidal nevus and identified the risk factors for transformation to melanoma [1]. Intraocular tumors are not easy to perform a diagnostic biopsy on, due to low diagnostic power and the risks of biopsy itself. Therefore, many oncologists are practically using these clinical risk factors for differential diagnosis [1]. However, even if a small pigmented choroidal lesion has known risk factors, it is difficult to determine with certainty the destructive treatments, such as enucleation or brachytherapy. However, observation alone can also be dangerous. Here, we report the cases of small choroidal lesions with high risk factors treated with intravitreal bevacizumab, which was developed as an anti-cancer drug [2], and transpupillary thermotherapy (TTT), which was a relatively simple method to perform at the outpatient clinic.

Subretinal fluid (SRF) associated with choroidal nevus is a risk factor for malignant transformation [1] and also an indication for treatment, as it leads to visual symptoms [3, 4]. For patients with this condition, treatment is required to prevent progressive loss of vision. There are various therapies to treat SRF, such as intravitreal anti-vascular endothelial growth factor (VEGF) injection [5–7], transpupillary thermotherapy (TTT) [8], and photodynamic therapy (PDT) with verteporfin [9–11]; these methods have shown variable results.

Bevacizumab has been shown to be effective in reducing neurosensory detachment by reducing the vascular permeability in various ocular diseases, such as choroid-retinal vascular diseases [12–16] and choroidal tumors [17–19]. If choroidal neovascularization (CNV) is present, it can be easily assumed that intravitreal anti-VEGF therapy may be helpful. However, in this study, we have investigated the effectiveness of anti-VEGF on subretinal fluid of small pigmented choroidal lesion “without CNV.”

With the expectation of reducing tumor size and SRF, we administered intravitreal bevacizumab injection (IVB) as a treatment for symptomatic subfoveal fluid (SFF) associated with small pigmented choroidal lesions. For the same subjects and with the same purposes, we also tried TTT as another treatment modality. TTT has been suggested as one of the therapeutic strategies for SRF associated with choroidal tumors [8, 17, 20].

In this study, we investigated the efficacy of IVB and TTT in resolving SFF-associated small pigmented choroidal lesions, and examined the relationship between treatment response and future tumor growth. Moreover, we described an unexpected effect of IVB on tumor growth.

## Methods

We retrospectively reviewed medical records of all patients who were diagnosed with small pigmented choroidal lesions at the Yonsei University Eye and ENT Hospital, between December 2005 and December 2016. Our study was approved by the Institutional Review Board at Yonsei University Medical Center (Reference No. 4–2017-0183) before data review, and it also adhered to the tenets of the Declaration of Helsinki.

Consecutive patients with small pigmented choroidal lesions (posteriorly located, apical height < 3 mm, and largest basal diameter [LBD] < 12 mm, Stage 1 by American Joint Commission on Cancer [AJCC]) and associated symptomatic SFF who were treated with IVB and/or TTT were included in this study. Patients who did not undergo follow-up examination after the initial diagnosis were excluded. Eyes with pigmented choroidal lesions with associated choroidal neovascularization were also excluded from the study.

The following data were collected at the initial examination via fundus photography, ultrasonography, optical coherence tomography (OCT; Stratus OCT, Carl Zeiss, Dublin, CA or Spectralis HRA + OCT, Heidelberg Engineering, Heidelberg, Germany), and fluorescein angiography (FA; Heidelberg Retina Angiograph system, Heidelberg Engineering, Heidelberg, Germany): tumor size (LBD and thickness), symptoms, surface features (orange pigmentation, drusen, and retinal pigment epithelium [RPE] alteration), SRF, distance to foveola and optic disc margin, and presence of choroidal neovascularization (CNV). Baseline demographic data, including age and gender, were also recorded. The observation of tumor growth or decrease was defined as a change in size, of at least 0.3 mm in any dimension, which was determined by comparing fundus photographs and B-scan ultrasonography images.

All patients were treated with IVB and/or TTT for decreasing SFF. Using an aseptic technique, 1.25 mg of bevacizumab was injected 3.0 or 3.5 mm posterior to the limbus through the pars plana using a 30-gauge needle in the operating room. TTT was performed under topical anesthesia via dilated pupil. Patients were treated with an infrared diode laser at 810 nm using a slit-lamp biomicroscope delivery system. Each tumor was covered entirely with confluent laser spots, with the power ranging from 160 to 500 mW and spot size between 1200 and 3000 μm to induce a slight color change with 1 min of exposure at each spot. An area of 1 disc diameter (DD) around the foveola and 1 DD around the disc margin were spared during TTT in all cases. Before the treatment, IVB and TTT have been approved for the treatment of the SFF in all patients in advance.

Patients were followed up 1–2 month(s) after IVB or TTT, and additional treatments were administered depending on the persistence of SRF, as demonstrated by OCT. For patients with progressively growing tumors noted on fundus photography or B-scan ultrasonography, which was suspected to be choroidal melanomas, we recommended treatment with TTT, plaque brachytherapy, or enucleation.

Relationship between response of SFF to IVB, or to observation and tumor growth, was analyzed using the Fisher’s exact test. Statistical analyses were performed using IBM SPSS Statistics, version 23 (IBM Corp., Armonk, NY, USA).

Tumor volume and doubling time were calculated as follows [21].

$$ \mathrm{Tumor}\ \mathrm{volume}=\frac{\pi }{6}\times \mathrm{tumor}\ {\mathrm{LBD}}^2\times \mathrm{height} $$

Tumor doubling time = 0.301 × time from initial to final volume / (log _10_ final volume – log _10_ initial volume).

## Results

A total of 19 eyes of 19 patients were enrolled in this study. The mean age of patients was 45.05 ± 10.88 years (range, 23–67 years). The mean LBD was 5.88 ± 1.46 mm (range, 3.11–8.38 mm), and the mean height was 1.98 ± 0.57 mm (range, 0.45–2.86 mm). Ten patients (10/19; 52.6%) were women. The presence of orange pigment was observed in 12 cases (63.1%). The mean distance to the foveola was 0.84 ± 1.37 mm (range, 0–5 mm), and the mean distance to the optic disc was 1.69 ± 1.55 mm (range, 0–5 mm; Table 1). Demographics and tumor features (including the presence of growth) of these 19 patients are summarized in Table 1.

**Table 1:** Patient Demographics, Ocular Parameters, Tumor Growth and Outcomes of Intravtireal Bevacizumab Injection or Transpupillary Thermotherapy on Subfoveal Fluid Associated with Small Pigmented Choroidal Lesions

Patient No.	Age range / Sex	Primary therapy #No.	SFF resolution	Additional therapy	Orange pigment	Foveal distance (mm)	Disc distance(mm)	Initial BCVA (Snellen)	BCVA change, Last BCVA (Snellen)	FU (mo)	Initial LBD (mm)	Initial height (mm)	Tumor growth, Last LBD (mm) *Last height (mm)
1	50–60/F	IVB #3	None	Refused	Yes	5	3.1	20/40	Worse, HM	79	7.4	2.8	↑↑↑, 9.34*10.1
2	40–50/F	IVB #6	None	Brachy Tx.	Yes	0	2.8	20/50	Worse, HM	18	6.12	1.94	↑↑↑, 8.16*5.22
3	50–60/M	IVB #2	Complete	Enucleation	Yes	0	0	20/200	Worse, LP-	48	5.29	2.06	↑↑↑, 7.76*3.87
4	30–40/M	IVB #2	None		Yes	0	2.2	20/50	Stable	68	5.59	1.64	→
5	50–60/M	IVB #2	None (Spontaneous resolution after 30 mo Obs.)		Yes	1.6	0	20/40	Improved, 20/20	59	4.4	2.08	→
6	30–40/F	IVB #2	None (Spontaneous resolution after 15 mo Obs.)		Yes	0	2.4	20/25	Worse, 20/63	19	3.11	1.28	→
7	30–40/M	IVB #1	Complete		No	2.1	5	20/32	Improved, 20/20	74	8.38	2.86	→
8	60–70/F	IVB #4	Partial		No	0	1.6	20/63	Stable	36	4.1	0.45	→
9	30–40/F	IVB #4	Partial		Yes	0	0	20/25	Worse, 20/40	39	5.88	2.38	→
10	40–50/F	IVB #3	None	TTT #2	Yes	0	1.8	20/40	Worse, 20/63	72	6.25	1.92	↑, 6.67*2.62
11	60–70/F	IVB #1	None	TTT #3 / Brachy Tx.	Yes	0.5	0	20/63	Worse, 20/125	51	6.81	2.23	↑, 7.24*2.52
12	30–40/F	(1) IVB #1 (3) IVB #2 (5) IVB #2	None	(2)TTT #2 (4)TTT #1 / Brachy Tx.	Yes	0	0	20/100	Worse, HM	42	7.11	1.91	↑↑↑, 10.63*4.12
13	40–50/F	IVB #2	None	TTT #3 / Brachy Tx.	No	1.1	1.1	20/25	Worse, 20/200	11	7.74	2.17	↑, 9.08*4.09
14	30–40/M	IVB #3	Partial	TTT #2	No	0.8	3.6	20/200	Stable	54	6.2	1.1	→
15	20–30/M	TTT #4	Complete		Yes	0	3	20/63	Improved, 20/25	86	7.17	2.24	↓, 3.96*0.7
16	50–60/M	TTT #2	Complete		No	0.75	0	20/40	Improved, 20/20	30	4	1.9	↓, 3.94*1.38
17	40–50/F	TTT #1	Partial		No	0	0	20/200	Stable	22	7.1	1.96	→
18	30–40/M	TTT #1	Complete		No	3.5	2.7	20/63	Improved, 20/40	3	4.72	2.42	Very short FU
19	50–60/M	Obs.	Spontaneous resolution		Yes	0.6	2.9	20/40	Stable	36	4.33	2.24	→

*IVB* intravitreal bevacizumab injection, *TTT* transpupillary thermotherapy, *No.* number, *SFF* subfoveal fluid, *BCVA* best corrected visual acuity, *FU* Follow-up duration, *mo* months, *LBD* largest base diameter, *M* male, *F* female, *Obs.* Observation, *Tx.* Therapy, *HM* hand motion, *LP*- no light perception

Of the 19 patients who had symptomatic SFF with decreased visual acuity, 14 were treated with IVB and four with TTT as the primary therapy, while one patient did not undergo any treatment. Five of the 14 patients who received IVB as the primary therapy were treated with TTT as a secondary therapy. IVBs were administered on an average of 2.86 ± 1.46 times (range, 1–6). TTT was applied on an average of 2.6 ± 0.55 sessions (range, 2–3) and 2.0 ± 1.41 sessions (range, 1–4) as the primary and secondary therapies, respectively.

Outcome measures included resolution of SFF and changes in visual acuity. Complete resolution of SFF was achieved in two eyes (2/14; 14.3%) after IVB, and in three eyes (3/4; 75%) after TTT. Partial resolution of SFF occurred in three eyes (3/14; 21.4%) after IVB, and in one eye (1/4; 25%) after TTT. All eyes showed a response to TTT, whereas nine eyes (9/14; 64.3%) that received IVB showed no improvement. Of these nine eyes, spontaneous resolution of SFF occurred in two eyes, 30 and 15 months after the last IVB (patient 5 and 6). One patient showed spontaneous resolution under observation without any treatment (patient 19; Tables 1 and 2).

**Table 2:** Treatment Outcomes to Intravitreal Bevacizumab Injection and Transpupillary Thermotherapy Regarding Response of Subfoveal fluid and Visual Acuity

SFF; BCVA	IVB	TTT
Complete resolution; Improved, No.(%)	2 (14.3%); 2 (22%)	3 (75%); 3 (75%)
Partial resolution; Stable, No.(%)	3 (21.4%); 2 (22%)	1 (25%); 1 (25%)
No change; Worsen, No.(%)	9 (64.3%); 5 (55.6%)	0 (0%); 0 (0%)
Total (No.)	14; 9	4; 4

*SFF* subfoveal fluid, *BCVA* best corrected visual acuity, *IVB* intravitreal bevacizumab injection, *TTT* transpupillary thermotherapy, *No.* number

Among 14 patients who received IVB as the primary therapy, five underwent TTT as an additional treatment. Following TTT, four out of these five patients (4/5; 80%) demonstrated an additional advantage. Complete resolution of SFF occurred in two eyes (2/5; 40%), and partial resolution occurred in two eyes (2/5; 40%; Table 3).

**Table 3:** Assessment of Treatment Response in the Patients Receiving Additional TTT after IVB as Primary Treatment for Subfoveal Fluid

No.	Age range / Sex	1st Tx. #No.	SFF 1st Response	2nd Tx. #No.	SFF 2nd Response	Additional therapy	Pre IVB SFF height (μm)	Post IVB, pre TTT SFF height (μm)	Post TTT SFF height (μm)	Tumor growth
10	40–50/F	IVB#3	None	TTT #2	Complete		114	167	0	↑
11	70–80/F	IVB#1	None	TTT #3	Partial	Brachy Tx.	380	390	54	↑
13	40–50/F	IVB#2	None	TTT #3	Partial	Brachy Tx.	292	671	315	↑
14	40–50/M	IVB#3	Partial	TTT #2	Complete		265	116	0	→
12	40–50/F	(1) IVB#1 (3) IVB#2 (5) IVB#2	None	(2) TTT #2 (4) TTT #1	None	Brachy Tx.				↑

*IVB* intravitreal bevacizumab injection, *TTT* transpupillary thermotherapy, *No.* number, *Tx.* Therapy, *1st* primary, *2nd* secondary, *SFF* subfoveal fluid, *M* male, *F* female

Of the nine eyes that received IVB alone, visual acuity improved in two eyes (2/9; 22%), remained stable in two eyes (2/9; 22%), and worsened in five eyes (5/9; 56%). Of the eyes that underwent TTT alone, visual acuity improved in three eyes (3/4; 75%), and remained stable in one eye (1/4; 25%)(Table 2).

**Fig. 1:**
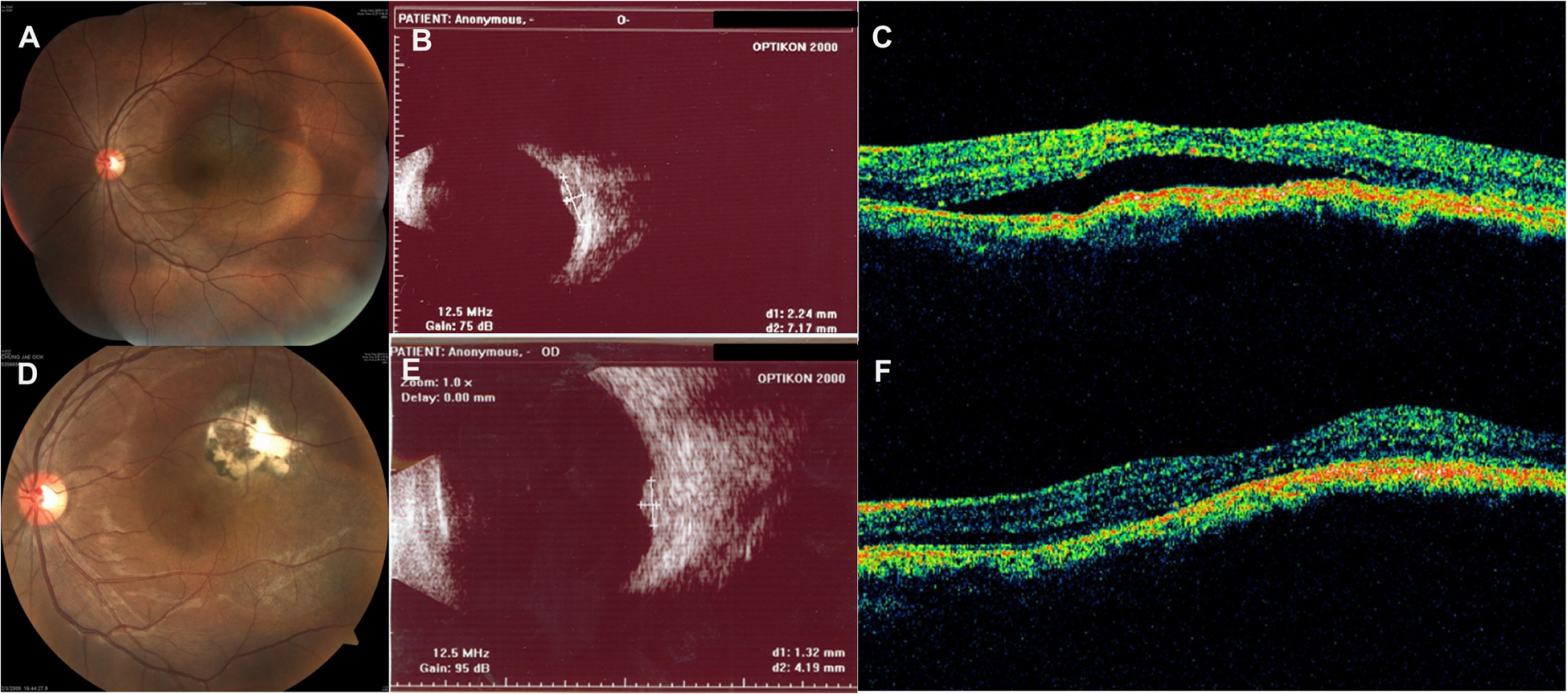
Treatment results of transpupillary thermotherapy for subfoveal fluid associated with choroidal melanocytic lesions. A 23-year-old man (Case 15) presented with 2-year history of decreased visual acuity of the left eye. His initial best corrected visual acuity (BCVA) was 20/63. **a** There was a pigmented subretinal mass supero-temporal to the fovea, **b** with a height of 2.24 mm and base diameter of 7.17 mm, as measured by B-scan ultrasonography. **c** Initial optical coherence tomography showed subretinal fluid at the macula. **f** After four sessions of transpupillary thermotherapy, subretinal fluid completely resolved and BCVA improved to 20/25. **d** The tumor showed scarring changes, and (**e**) tumor size decreased to 1.32 mm (height) by 4.13 mm (base diameter), as measured by B-scan ultrasonography. Improved vision and attached retinal status were maintained for 46 months after the last treatment. Tumor size further decreased to 0.50 mm (height) by 4.18 mm (base diameter)

Despite the small number of enrolled patients, TTT showed better effects than IVB, in terms of fluid reduction and visual improvement. Figure 1 depicts the findings of a patient who showed complete resolution of SFF, improved visual acuity, and reduced tumor size after TTT.

Among 15 eyes that received IVB or observation as the primary therapy, tumor growth was detected in seven eyes (7/15; 46.7%) (patients 1, 2, 3, 10, 11, 12, and 13). Of the patients who underwent TTT alone, none of them demonstrated tumor growth (0/4, 0%). IVB was not effective in inhibiting tumor growth.

Of the patients who were followed up for at least 11 months, seven patients (7/18, 38.9%) showed tumor growth during follow-up (mean, 46.9 months; range, 11–86 months). With the assumption of choroidal melanoma, we advised these seven patients with enlarged tumors to undergo further treatment. Four patients received ruthenium-106 brachytherapy (patients 2, 11, 12, and 13), one patient underwent TTT (patient 10), and one patient underwent enucleation (patient 3). One patient was advised to undergo brachytherapy, but he refused and was lost to follow-up (patient 1).

**Fig. 2:**
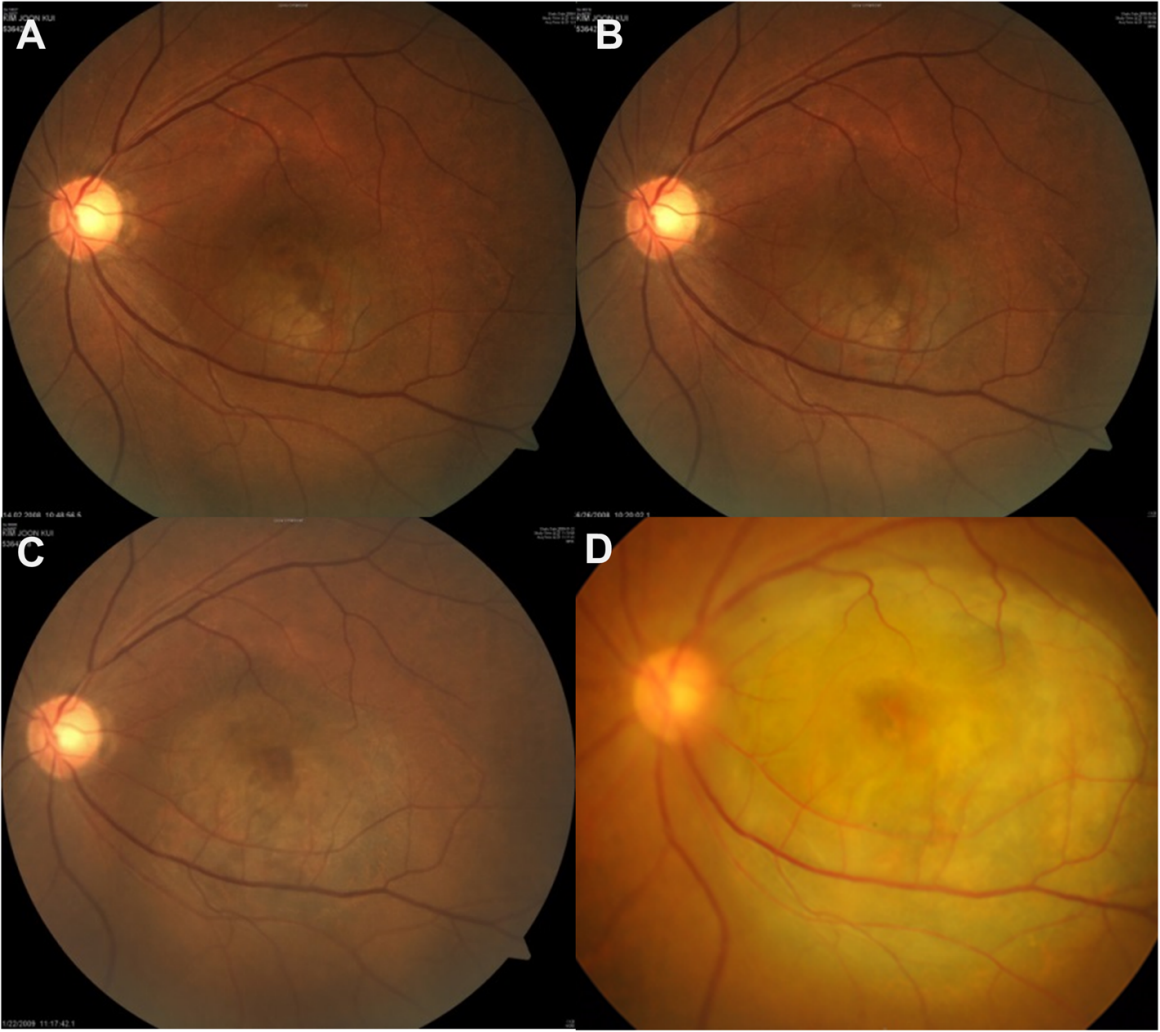
Potential adverse effect of intravitreal bevacizumab injection on tumor growth. A 58-year-old man (Case 3) presented with 1-month history of decreased visual acuity in the left eye. His initial best corrected visual acuity (BCVA) was 20/200. **a** There was a pigmented choroidal mass at the fovea, with a height of 2.06 mm and a base diameter of 5.29 mm, as measured by B-scan ultrasonography. Shallow subfoveal fluid was observed on optical coherence tomography. Calculated tumor volume was 30.18 mm^3^. Change in tumor size was monitored without any treatment. **b** After 4.5 months, tumor growth was not observed. Calculated tumor volume was 28.98 mm^3^, with a height of 2.17 mm and a base diameter of 5.05 mm. However, as subfoveal fluid persisted, intravitreal bevacizumab injections were administered twice at an interval of 1.5 months. **c** At 5 months after the last intravitreal injection, marked tumor growth was noted on B-scan ultrasonography and fundus photography. Calculated tumor volume was 66.29 mm^3^, with a height of 2.96 mm and a base diameter of 6.54 mm. Tumor-doubling time from the period before injections was 169.73 days. Subsequently, biopsy and treatment were recommended; however, the patient refused. **d** After 2 years, the tumor had grown to a size of 280.10 mm^3^. Tumor-doubling time was calculated as 197.32 days. Finally, the patient underwent enucleation

In fact, one patient showed accelerated tumor growth after IVBs (Fig. 2). This 58-year-old man had presented with a 1-month history of decreased visual acuity. A pigmented choroidal mass was noted at the fovea with a height of 2.06 mm and base diameter of 5.29 mm, as measured by B-scan ultrasonography and fundus photography. Calculated tumor volume was 30.18 mm^3^. Shallow SFF was observed on OCT. There was no change in tumor size over the 4.5-month follow-up period without any treatment. However, SFF persisted and IVBs were administered twice for treatment of the fluid. At 5 months after IVBs, prominent tumor growth was observed. Calculated tumor volume was 66.29 mm^3^, with a height of 2.96 mm and a base diameter of 6.54 mm. Subsequently, the rate of tumor growth showed further acceleration and eventually, enucleation had to be performed.

In this study, treatment response (or lack of response) to IVB may indicate choroidal melanoma or future progression to choroidal melanoma. Of the seven patients who had SFF refractory to IVB, six showed tumor growth during the follow-up period (6/7; 86%). When SFF showed a decrease in response to IVB or observation, there was no tumor growth in seven out of eight patients (7/8; 88%). In conclusion, therapeutic response to IVB was valuable in predicting subsequent tumor growth with statistical significance (Fisher’s exact test, *p* = 0.010; Table 4).

**Table 4:** Relationship between Therapeutic Response of Subfoveal fluid to Intravitreal Bevacizumab Injection or Observation and Tumor Growth

	Tumor growth	Tumor size stable	Total
SFF Decreased	1	7 (87.5%)	8
SFF Refractory	6 (85.7%)	1	7
Total	7	8	15

*SFF* subfoveal fluid

*p* = 0.010 (Fischer’s exact)

## Discussion

Bevacizumab, a recombinant humanized monoclonal antibody that inhibits the vascular endothelial growth factor (VEGF), was originally developed for the treatment of metastatic colon cancer, and is still under investigation for numerous other primary and metastatic cancers [2]. Moreover, bevacizumab has been shown to be effective in reducing neurosensory detachment related with choroidal tumors [17–19]. With the expectation of reducing tumor size and SFF, we administered intravitreal bevacizumab injection (IVB) as a treatment method for small pigmented choroidal lesions with symptomatic subfoveal fluid (SFF).

In pigmented choroidal lesions with symptomatic leakage, various treatments, such as laser photocoagulation [22–26], PDT with verteporfin [9–11, 25, 27–30], TTT [8], and intravitreal anti-VEGF injections [5–7], have been used; however, majority of them have been reported in patients with CNV.

There have been several reports of intravitreal anti-VEGF therapy for the treatment of SRF associated with choroidal nevi, all of which were used to treat SRF with co-existing CNV [5–7]. In these reports, intravitreal anti-VEGF injection was reported as effective. In the presence of CNV, it is easy to assume that SRF could be reduced by anti-VEGF effect. However, in this study, by excluding CNV-accompanying cases, we focused more on the effect of the tumor itself on fluid, rather than the CNV that is secondarily induced by the tumor. In our study, IVB showed relatively less effectiveness in patients with CNV-free small pigmented choroidal lesions. As IVB is primarily effective in neovascularization, the difference in these results may be due to the presence of CNV in pigmented choroidal lesions.

A previous study reported that subthreshold TTT was effective in the treatment of SFF associated with small pigmented choroidal lesions [8]. However, in that study, subjects who had previous focal photocoagulation were also included; moreover, the presence of CNV was not confirmed. Out of 13 total patients enrolled, 11 (84.6%) patients showed complete resolution of SRF, and nine (69.3%) patients maintained or showed improvement in the best corrected visual acuity. These results were similar to ours. In our study, we found that TTT was not only beneficial in patients without any previous therapy, but also in those who had previously undergone IVB therapy.

In addition to the purpose of reducing SRF by IVB, we also checked the effect of IVB in inhibiting tumor growth. In the present study, the number of enrolled patients was small, and there was no control group. However, it was evident that IVB could not effectively inhibit tumor growth. The non-beneficial effects of bevacizumab on choroidal melanomas have been reported before. Lima et al. showed a lack of benefit from bevacizumab in the inhibition of tumor progression in their study of three patients with choroidal melanoma who inadvertently received multiple IVBs [31].

In this paper, a case with no growth during observation period and marked increase in growth after IVB were reported. We were concerned, as such tumor growth could be a possible adverse effect of bevacizumab. Recently, similar cases of paradoxical enlargement were reported in patients with uveal melanoma after IVB [32]. They used IVB as a neoadjuvant concept for treating uveal melanoma, but observed that the tumor size increased rather than decreased, and the study was terminated early. It was also possible that the observation of continued growth was due to the natural history of uveal melanoma. However, they suggested the need for caution when using anti-VEGF agents for uveal melanomas.

In fact, since bevacizumab has been used to inhibit primary or metastatic cancer in a different type of cancers, such finding from one case is not easily understood intuitively. There have been conflicting reports on VEGF levels in uveal melanoma [33–36]. A possible mechanism for this paradoxical phenomenon is that alteration of VEGF, one of the most potent tumor angiogenic factors, by IVB may affect the dormancy, which is a distinctive feature of melanoma that differentiates it from other cancers. Uveal melanoma is characterized by slow progression and periods of dormancy (both primary and metastatic tumors). It has been suggested that this dormancy is associated with an avascular phase [37]. VEGF expression of tumor tissue may be related to the dormant status of uveal melanoma. Conversion to the angiogenic phenotype is due to an alteration in the balance of inhibitory and stimulatory factors [37]. Based on this possible mechanism of tumor growth related to IVB, it may be assumed that anti-VEGF therapy and its withdrawal influences the delicate balance among angiogenic factors, thus eventually breaking the dormancy of uveal melanoma. In addition, several previous experimental reports can support the hypothesis that anti-VEGF therapy may have a paradoxical effect, different from what is originally expected. In one notable study in the field of cancer biology, it has been experimentally demonstrated that the inhibition of angiogenesis pathway, such as VEGF, could alter the natural history of a tumor by increasing its invasion and metastasis [37, 38]. El Filali et al. described an accelerated tumor growth following IVB in murine B16 melanoma cell-containing eyes, and suggested possible adverse effects of bevacizumab on choroidal melanoma cells [36]. Of course, we observed the adverse effect in only one case, which could have happened by chance, and we should not mistakenly make any hasty generalization. However, IVB may have the potential adverse effect, even at a low probability. Overall, IVB should be used with caution in treatment of SRF associated with small pigmented choroidal lesion, due to its lower therapeutic effect and possible adverse effects.

In this study, treatment response (or lack of response) to IVB may indicate choroidal melanoma or future progression to choroidal melanoma (Fisher’s exact test, *p* = 0.010). In other words, therapeutic response to IVB can be used to diagnose or prognosticate malignancy. Small pigmented choroidal lesions with SRF that do not respond to IVB should be carefully observed.

Limitations of our study include a small sample size and lack of a control group. Since randomization has not been performed to IVB group and TTT group, comparison of the two treatment methods is not appropriate. In addition, generalization of a case showing paradoxical growth should be avoided.

## Conclusion

In conclusion, IVB was not very effective in terms of resolution of symptomatic SFF and improvement of BCVA associated with small pigmented choroidal lesions without CNV, and was unable to effectively inhibit tumor growth. Treatment response (or lack of response) to IVB may indicate choroidal melanoma or future progression to choroidal melanoma. Therefore, bevacizumab should be used with caution in pigmented choroidal lesions.

## Abbreviations

AJCC: American Joint Commission on Cancer
CNV: Choroidal neovascularization
DD: Disc diameter
FA: Fluorescein angiography
IVB: Intravitreal bevacizumab injection
LBD: Largest basal diameter
OCT: Optical coherence tomography
PDT: Photodynamic therapy
RPE: Retinal pigment epithelium
SFF: Subfoveal fluid
SRF: Subretinal fluid
TTT: Transpupillary thermotherapy
VEGF: Vascular endothelial growth factor
